# Troubles lipidiques et glucidiques à risque cardio-vasculaires chez les personnes vivant avec le virus d’immunodéficience humaine sous traitement antirétroviral: cas du centre de prise en charge médicale de l’ONG Espoir-Vie-Togo à Lomé

**DOI:** 10.11604/pamj.2019.34.203.20600

**Published:** 2019-12-18

**Authors:** Djagadou Kodjo Agbeko, Tchamdja Toyi, Djalogue Lihanimpo, Némi Komi Dzidzonu, Kaaga Laconi, Balaka Abago, Djibril Mohaman Awalou

**Affiliations:** 1 Service de Médecine Interne, Faculté des Sciences de la Santé, Université de Lomé, Togo

**Keywords:** Traitement ARV, PVVIH, troubles métaboliques, Lomé, ARV treatment, PLHIV, metabolic disorders, Lomé

## Abstract

Les médicaments antirétroviraux sont responsables d’effets secondaires ou indésirables. Ils peuvent comporter une redistribution du tissu adipeux, des troubles du métabolisme lipidique ou glucidique. Il s’avère nécessaire, en face du nombre croissant de personnes vivant avec le VIH (PVVIH) mises sous traitement antirétroviral d’évaluer la fréquence des troubles métaboliques glucidique et lipidique chez les patients qui sont sous traitement antirétroviral (ARV). L’analyse a porté sur 493 patients infectés par le VIH/SIDA et qui sont sous ARV pris en charge dans le centre de prise en charge médicale de l’ONG Espoir-Vie-Togo à Lomé. Les données paracliniques telles la glycémie, les taux sériques de cholestérol total, de triglycérides, de HDL-c et LDL-c ont été étudiés. L’étude a montré que les anomalies suivantes: l’hypercholestérolémie, l’hyper LDL-cholestérolémie, l’hypo HDL-cholestérolémie ont été retrouvées respectivement chez 41,4%, 23,5% et 17,4% des patients. La fréquence de l’hyperglycémie était de 12,4%. Il est à noter que la fréquence des troubles lipido-glucidiques était plus élevée chez les patients sous les schémas comprenant les inhibiteurs de protéases. A ces troubles s’ajoute une fréquence de 31,2% de patients en situation de surpoids ou d’obésité déclarée. Les troubles du métabolisme lipidique et glucidique existent dans cette population. La fréquence de ces troubles diffère selon que le patient soit sous une trithérapie comprenant les inhibiteurs de protéase ou non.

## Abstract (en)

Antiretroviral drugs are responsible for side effects or undesirable effects. These may include redistribution of adipose tissue and disorders of the lipid or carbohydrate metabolism. Given the growing number of people living with HIV (PLHIV) on antiretroviral therapy, it is necessary to assess the frequency of disorders of carbohydrate and lipid metabolism in patients who are on antiretroviral therapy (ARV). Our analysis focused on 493 patients with HIV/AIDS and on ARV treated in the Medical Centre of the ONG Espoir Vie Togo, Lomé. Paraclinical data such as blood glucose, serum total cholesterol, triglycerides, HDL cholesterol and LDL cholesterol were studied. The study showed the following anomalies: hypercholesterolemia, LDL hypercholesterolemia, HDL-hypocholesterolemia were found in 41.4%, 23.5% and 17.4% of patients respectively. The incidence of hyperglycemia was 12.4%. It is important to note that the incidence of lipido carbohydrate disorders was higher in patients receiving treatment schedule including protease inhibitors. The study also highlighted that 31.2% of patients with disorders of carbohydrate and lipid metabolism were overweight or obese. The incidence of these disorders differs depending on whether patients were under triple therapy including protease inhibitors or not.

## Introduction

Face à l’épidémie du VIH/Sida, qui constitue un défi majeur en santé publique, le développement des médicaments antirétroviraux (ARV) pouvant freiner la propagation du VIH a été l’un des plus rapides de l’histoire. Ainsi à partir de 1996, la mise sur le marché d’ARV puissants a transformé cette maladie à issue fatale en maladie chronique, mais de nouvelles problématiques ont émergé. L’optimisme suscité par les réels progrès des ARV a été rapidement tempéré par leurs effets secondaires. Les ARV présentent tous une certaine toxicité pour l’organisme. Ils sont responsables d’effets secondaires ou indésirables, plus ou moins marqués à court, moyen et long termes. Ils peuvent comporter une redistribution du tissu adipeux, des troubles du métabolisme lipidique ou glucidique [1, 2]. En Afrique, diverses études réalisées ont permis de faire le même constat de l’apparition de troubles métaboliques chez les patients vivants avec le VIH lors de la mise sous traitement [3-6]. Il nous a paru nécessaire de réaliser cette étude préliminaire dans ce centre de prise en charge médicale en ambulatoire des PVVIH.

## Méthodes

Il s’est agi d’une étude descriptive et transversale portant sur les dossiers de patients suivis dans le centre de prise en charge médicale de l’ONG Espoir-Vie-Togo à Lomé. Elle s’est déroulée du 1^er^ janvier au 31 décembre 2010. Ont été inclus dans l’étude, les patients séropositifs au VIH et qui ont été mis sous traitement depuis 12 mois au moins. Tous les patients présentant déjà à l’inclusion du traitement un trouble métabolique lipidique et/ou glucidique n’ont pas été inclus dans l’étude. Pour chaque patient, les paramètres suivants ont été analysés: l’identité des patients: âge, sexe, domicile et statut matrimonial; les données cliniques: poids, taille, indice de masse corporelle (IMC), le type de VIH; les données paracliniques, essentiellement les résultats des bilans de suivi composés de: taux de CD4, glycémie, cholestérolémie totale, triglycéridémie, HDL-cholestérol et LDL-cholestérol; les données thérapeutiques des patients: combinaison du traitement.

Les examens paracliniques ont été réalisés en utilisant les mêmes appareils de biologie médicale. Les troubles lipidiques et glucidiques à risques cardio-vasculaires étudiés étaient l’hypercholestérolémie totale, l’hypertriglycéridémie, l’hyperglycémie, l’hypo HDL cholestérolémie, et l’hyper LDL cholestérolémie. L’hypercholestérolémie totale a été définie par le dosage du cholestérol total supérieur ou égal à 2g/l. L’hypertriglycéridémie a été définie par les triglycérides sanguins supérieurs ou égaux à 1,60g/l. De même l’hypoHDL cholestérolémie et l’hyperLDL cholestérolémie ont été définis respectivement pas des taux de HDL cholestérol et de LDL cholestérol respectivement inférieurs ou égaux à 0,40g/l et supérieurs ou égaux à 1,60g/l. Enfin, on a parlé d’hyperglycémie si la glycémie était supérieure ou égale 1,26g/l.

## Résultats

Nous avons enregistré 493 sujets dont la durée moyenne de traitement ARV a été de 14 mois.

### Description de l’échantillon

Nous avions noté une prédominance féminine (78,3%) avec un sex-ratio à 3,6. L’âge moyen a été de 38,84 ± 10,87 ans et les extrêmes étaient de 3 ans et 74 ans. Les tranches 30 à 39 et 40 à 49 ont été prédominantes (70,5%). La majorité (99,4%) des patients étaient infectés par le VIH-1 et peu de patients par le VIH-2 (p = 0,01). Les patients avaient un IMC moyen de 23,74 ± 4,87kg/m2. Une proportion importante des patients soit 31,2% était en situation de surpoids et d’obésité déclarée. Sur le plan immunologique, la majorité des patients (78,4%) avaient un taux de lymphocytes CD4 inférieur à la normale (p = 0,01). La moyenne de cellules CD4 étant de 370,67 cells/µl. Enfin, s’agissant de la thérapeutique, la majorité soit 86,8% des patients était sous la combinaison thérapeutique associant 2 inhibiteurs nucléosidiques de la transcriptase inverse et 1 inhibiteur non nucléosidique de la transcriptase inverse (p = 0,01).

### Troubles lipidiques et glucidiques

Parmi nos patients, 41,4% avait une hypercholestérolémie élevée. De même on a noté que 12,7% des patients avaient un taux de triglycérides élevé. Pour ce qui du HDL-cholestérol, 17,4% des patients avaient un taux de HDL-Cholestérol inférieur à la normale. Le taux moyen de cholestérol HDL est de 0,60 ± 0,19 g/l et a pour valeurs extrêmes 0,24 à 1,09g/l. La différence des fréquences entre taux normal et élevé est très significative (p = 0,01). Enfin, 23,5% avaient un taux supérieur à la normale. Le taux moyen de cholestérol LDL est de 1,18 ± 0,46g/l. Enfin, on a noté une hyperglycémie chez 12,4% des patients de notre étude.

### Troubles lipidiques et trithérapie

Le taux de cholestérol total était élevé 40,0% et 41,8% respectivement selon que le traitement comprenne l’inhibiteur de la protéase (IP) ou non (Tableau 1). L’hypertriglycéridémie a été notée chez respectivement 17,8% et 12,0% des patients sous traitement IP ou non (Tableau 2). Nous avons aussi retrouvé un taux bas de HDL-cholestérol respectivement chez 18,2% et 17,2% des patients selon que le patient soit sous traitement IP ou non (Tableau 3). Pour ce qui est du LDL-cholestérol, respectivement chez 27,3% et 22,8% des patients selon que le patient soit sous traitement IP ou non (Tableau 4).

**Tableau 1:** Répartition des patients en fonction de la cholestérolémie totale et de la trithérapie

	Moins de 0,90 *(*%*)*	De 0,90 à 2,00 *(*%*)*	*Plus de 200 (*%*)*	Total *(*%*)*
**2INTI + 1INNTI**	32 *(7,6)*	212*(50,6)*	175 *(41,8)*	419 *(100,0)*
**2INTI + 1IP**	9 *(13,8)*	30 *(46,2)*	26 *(40,0)*	65 *(100,0)*

*INTI = inhibiteurs nucléosidique de la transcriptase inverse; *INNTI= inhibiteurs non nucléosidique de la transcriptase inverse; *IP= l’inhibiteur de la protéase

**Tableau 2:** Répartition des patients selon le taux de triglycérides et de la trithérapie

	Moins de 0,50 (%)	De 0,50 à 1,60 (%)	Plus de 1,60 (%)	Total (%)
**2INTI + 1INNTI**	20 *(4,9)*	340 *(83,1)*	49 *(12,0)*	409 *(100,0)*
**2INTI + 1IP**	2 *(3,2)*	49 *(79,0)*	11*(17,8)*	62 *(100,0)*

*INTI = inhibiteurs nucléosidique de la transcriptase inverse ; *INNTI= inhibiteurs non nucléosidique de la transcriptase inverse; *IP= l’inhibiteur de la protéase

**Tableau 3:** Répartition des patients en fonction de HDL-c et de la trithérapie

	Moins de 0,40 (%)	De 0,40 à 0,60 (%)	Plus de 1,60 (%)	Total (%)
**2INTI + 1INNTI**	10 (17,2)	17(29,3)	31 (53,5)	58 (100,0)
**2INTI + 1IP**	2 (18,2)	3 (27,3)	6 (54,5)	11 (100,0)

*INTI = inhibiteurs nucléosidique de la transcriptase inverse; *INNTI= inhibiteurs non nucléosidique de la transcriptase inverse; *IP= l’inhibiteur de la protéase

**Tableau 4:** Répartition des patients en fonction de LDL-c et de la trithérapie

	Moins de 1,60 (%)	*1,60 et plus* (%)	Total (%)
**2INTI + 1INNTI**	44 *(77,2)*	13 *(22,8)*	57 *(100,0)*
**2INTI + 1IP**	8 *(72,7)*	3 *(27,3)*	11 *(100,0)*

*INTI = inhibiteurs nucléosidique de la transcriptase inverse; *INNTI= inhibiteurs non nucléosidique de la transcriptase inverse; *IP= l’inhibiteur de la protéase

### Troubles glucidiques et trithérapie

Nous avons noté une fréquence de glycémie élevée chez respectivement 9,7% et 12,8% selon que le patient soit sous traitement IP ou non (Tableau 5).

**Tableau 5:** Répartition des patients selon la glycémie et de la trithérapie

	Moins de 0,70 *(%)*	De 0,70 à 1,10 *(%)*	Plus de 1,10 *(%)*	Total *(%)*
**2INTI + 1INNTI**	17 *(4,1)*	348 *(83,1)*	54 *(12,8)*	419 *(100,0)*
**2INTI + 1IP**	6 *(9,7)*	50 *(80,6)*	6 *(9,7)*	62 *(100,0)*

*INTI = inhibiteurs nucléosidique de la transcriptase inverse; *INNTI= inhibiteurs non nucléosidique de la transcriptase inverse; *IP= l’inhibiteur de la protéase

## Discussion

### Données sociodémographiques

L’âge des patients variait entre 3 et 74 ans. L’âge moyen des patients était de 38,84 ans. Zouiten *et al.* [7] dans une étude réalisée entre 1986 et 2003 en Tunisie ont trouvé une tranche d’âge variant entre 16 mois à 69 ans et une moyenne d’âge de 35,7 ans. Les tranches 30 à 39 et 40 à 49 étaient les plus représentées et occupent à elles seules 70,5% de notre population d’étude. Okome Nkoumou *et al.* [8] dans leur étude avaient trouvé que 62,6% des patients étaient de la tranche d’âge 20 et 50 ans. L’adulte jeune semble le plus touché puisque 80,0% des patients ont un âge compris entre 20 et 50 ans. Cette situation s’explique par deux raisons: l’âge jeune de la population générale d’une part et d’autre part la tranche d’âge 20-50 ans est l’âge d’activité sexuelle [9]. Pour notre série, la prédominance féminine (78,3%) a été significative avec un sex-ratio femme-homme de 3,6. Cette prédominance des patients du sexe féminin est nettement supérieure aux données de l’étude d’Okome Nkoumou *et al.* [8] qui avaient trouvé une prédominance du sexe féminin à 54,7% soit un sex-ratio homme-femme de 0,8. Ces données montrent aussi la prédominance du sexe féminin dans l’infection au VIH. Cette dominance du sexe féminin serait liée d’une part à l’anatomie de l’appareil génital féminin qui est plus exposé à l’infection au VIH. D’autre part le dépistage du VIH est plus important chez les femmes que chez les hommes. Car les femmes acceptent plus facilement la réalisation du test; de plus, la surveillance sentinelle du VIH auprès des femmes enceintes initiée dans le cadre de la Prévention de la Transmission du VIH de la Mère à l’Enfant (PTME) contribue au dépistage d’un nombre important de femmes. Le VIH-1 est majoritaire dans notre étude soit 51,5% contre seulement 2 cas (soit 0,8%) pour le VIH-2 et 1 cas (soit 0,4%) pour la double séropositivité. Le type de VIH est non précisé pour 47,9%. Les résultats de notre série sont différents de ceux obtenus par Okome Nkoumou *et al*.[8] la quasi-totalité (99,6%) des patients était infectée par le VIH-1.

### Aspects thérapeutiques

Les 493 patients de notre série étaient sous traitement ARV. Ils ont tous bénéficié d’une trithérapie: 86,8% était sous le protocole 2INTI + 1INNTI et 13,2% sous le protocole 2INTI + 1IP. Ces résultats sont similaires à ceux de Okome Nkoumou *et al.* [8] qui montraient que 72,9% des patients bénéficiaient d’un schéma thérapeutique 2INTI + 1INNTI et 17,2% d’un schéma 2INTI + 1IP. Plusieurs études ont montré que l’association 2INTI + 1INNTI avait une efficacité similaire à l’association de 2INTI + 1IP. Le choix thérapeutique initial est une décision essentielle pour l’avenir thérapeutique du patient. Le choix de la combinaison thérapeutique initiale tient souvent compte de plusieurs facteurs notamment de la disponibilité des molécules, le type de protocole adopté par chaque pays et de la situation particulière de chaque patient: l’existence d’anomalies biologiques, les traitements antituberculeux ou d’autres thérapies susceptibles d’interférer avec les ARV.

### Aspects immunologiques

Selon nos résultats, la moyenne de cellules CD4 était de 370,67 cells/µl. Carr A *et al.* [8] ont trouvé une moyenne des lymphocytes CD4 qui variait entre 450 et 581 cells/µl selon que les personnes utilisaient des combinaisons incluant les IP ou non [4]. Le taux de lymphocytes CD4 a montré une ascension supérieure à 500 cells/µl seulement chez 21,6% des patients. Zouiten *et al*. [7] avaient trouvé 28,2%. Selon Zouiten F *et al.* [7], l’absence d’amélioration nette du statut immunitaire chez les patients s’explique par le fait que la majorité d’entre eux étaient au stade SIDA de la maladie à l’initiation du traitement et avaient un taux de CD4 inférieur à 200 cells/µl. La restauration du statut immunitaire est jugée sur l’évolution du taux de CD4 dont la remontée est plus lente lorsque le traitement a été introduit tardivement [10].

### Profil lipidique de la population d’étude

#### Cholestérolémie totale

L’hypercholestérolémie est observée chez 41,4% des patients de l’étude. Friis-Moller *et al.* [11] en 2003 dans leur étude DAD sur les classes d’ARV et le risque d’infarctus du myocarde, ont trouvé que 21,1% des patients sous ARV avait une hypercholestérolémie. Les patients de notre série dont la durée moyenne de traitement a été de 14 mois ont vu leur état immunitaire amélioré et ont eu le temps de subir les effets secondaires des ARV. L’hypercholestérolémie est observée chez 26 patients utilisant le protocole 2INTI + 1IP soit 40,0% de patients sous ce protocole. L’hypercholestérolémie a été notée chez 41,8% des patients sous le protocole 2INTI + 1INNTI. Friis-Moller *et al*. [11] dans une étude sur l’association entre dyslipidémie et schéma d’ARV connus, ont trouvé 27,0% et 22,8% de patients respectivement sous protocole 2INTI + 1IP et 2INTI + 1INNTI. Cette différence pourrait être liée à la durée d’exposition au schéma ARV car le taux de cholestérol total est stable après 3 à 6 mois de traitement antirétroviral.

#### Triglycéridémie

L’hypertriglycéridémie est observée chez 12,7% des patients. Friis-Moller *et al*. [11] ont trouvé 32,2% d’hypertriglycéridémie. Le Groupe d’Evaluation Cœur et Muscle a trouvé que 15 à 40% des patients sous traitement avait une hypertriglycéridémie (GECEM, 2005) [12]. Dans notre étude, selon le type de schéma thérapeutique, la fréquence de l’hypertriglycéridémie est respectivement de 17,8% et 12,0% chez les patients sous les schémas 2INTI + 1IP et 2INTI + 1INNTI. Friis-Moller *et al*. [11] dans une autre étude ont trouvé que 40,0% et 31,8% des patients respectivement sous les schémas 2INTI + 1IP et 2INTI + 1INNTI avaient une hypertriglycéridémie. La faible fréquence d’hypertriglycéridémie pourrait être liée au fait que la hausse du taux sérique de triglycérides du début du traitement a commencé une régression et une stabilisation en raison de la bonne réponse du traitement ARV [13].

#### Cholestérolémie HDL

L’hypocholestérolémie HDL est observée chez 17,4% de nos patients. Friis-Moller *et al*. [11] en 2003 ont trouvé que 26,1% des patients avaient un taux bas de cholestérol HDL. Selon le schéma thérapeutique, l’hypocholestérolémie HDL est notée chez 18,2% et 17,2% de patients respectivement sous les schémas 2INTI + 1IP et 2INTI + 1INNTI. Friis-Moller *et al.* [11] ont trouvé un taux sérique de cholestérol HDL bas chez 27,1% (2INTI + 1IP) et 19,1% (2INTI + 1INNTI) des patients. De façon générale, l’hypocholestérolémie HDL est multifactorielle notamment la durée d’exposition, le type d’ARV et les prédispositions génétiques.

#### Cholestérolémie LDL

L’hypercholestérolémie LDL est observée chez 23,5% des patients. Cette fréquence a été de 27,3% et 22,8% selon que le patient soit sous traitement comprenant un IP ou non. Cette fréquence du taux élevé de LDL-cholestérol bien que relativement faible dans la population d’étude est inquiétante car 27,3% des patients sous IP sont concernés. En effet, ce dernier est à l’origine de la formation de plaques d’athérome et donc l’augmentation du risque cardiovasculaire. L’infection par le VIH engendre par elle-même des dyslipidémies. Le traitement anti-VIH inverse, au moins partiellement, les effets du virus mais introduit une grande complexité dans l’efficacité des différents antirétroviraux sur les lipides. L’impact global des antirétroviraux sur les lipides chez un patient donné dépend de l’effet d’exposition et/ou de la durée d’exposition. Il dépend également des conditions de l’hôte, notamment son style de vie et l’alimentation, des prédispositions génétiques, ainsi que de ses comorbidités telles l’insulinorésistance et le diabète [13]. La hausse du taux de cholestérol total, de LDL-cholestérol, des triglycérides et la baisse du taux de HDL-cholestérol sont des marqueurs établis de l’augmentation du risque vasculaire. Ce sont donc des problèmes clés qui augmentent le risque de troubles cardiovasculaires [14].

### Profil glycémique

L’hyperglycémie est observée chez 12,4% de nos patients. Le Groupe d’Evaluation Cœur et Muscle a trouvé une fréquence d’environ 40% de diabétiques et de patients insulinorésistants (GECEM, 2005). La fréquence de l’hyperglycémie chez les patients sous traitement IP est de 9,7%. Passalaris *et al.* [15] ont trouvé une fréquence de 25 à 60% d’insulinorésistance chez les patients recevant des IP. Cette faible fréquence d’hyperglycémie pourrait s’expliquer par la durée d’exposition et le type d’ARV d’une part, la faible prédisposition des patients au diabète d’autre part. Selon Hadigan *et al*. [16], la fréquence des anomalies de la tolérance au glucose augmente chez les hommes sous traitement IP. Les patients traités par les IP doivent donc adopter les mesures préventives d’une hyperglycémie: la pratique d’activités physiques régulières et la diminution de la consommation de sucres d’absorption rapide.

## Conclusion

Notre étude a porté sur l’évaluation de la fréquence des troubles métaboliques en l’occurrence les troubles glucidiques et lipidiques au sein des personnes vivant avec le VIH en milieu hospitalier au Togo. L’hypercholestérolémie culmine les troubles du métabolisme lipidique avec une fréquence de 41,4% suivi de l’hyper LDL-cholestérolémie (23,5%), de l’hypo HDL-cholestérolémie (17,4%) et d’hypertriglycéridémie (12,7%). La fréquence de l’hyperglycémie est de 12,4%. La fréquence de ces troubles diffère selon que le patient soit sous une trithérapie comprenant les inhibiteurs de protéase ou non. Le VIH/SIDA étant un facteur de risque cardiovasculaire, il est donc judicieux de faire un suivi clinique et biologique des patients souffrant du VIH afin de dépister tôt les éventuels facteurs de risques.

### Etat des connaissances actuelles sur le sujet

- Les médicaments antirétroviraux présentent tous une certaine toxicité pour l’organisme;
- Ils sont responsables d’effets secondaires ou indésirables, plus ou moins marqués à court, moyen et long termes;
- Ils peuvent comporter une redistribution du tissu adipeux, des troubles du métabolisme lipidique ou glucidique.

### Contribution de notre étude à la connaissance

- Notre étude a montré que les anomalies suivantes: l’hypercholestérolémie, l’hyper LDL-cholestérolémie, l’hypo HDL-cholestérolémie ont été retrouvées respectivement chez 41,4%, 23,5% et 17,4% des patients;
- La fréquence de l’hyperglycémie était de 12,4%; il est à noter que la fréquence des troubles lipido-glucidiques était plus élevée chez les patients sous les schémas comprenant les inhibiteurs de protéases ;
- A ces troubles s’ajoute une fréquence de 31,2% de patients en situation de surpoids ou d’obésité déclarée; il est donc indispensable de faire un suivi clinique et biologique des patients souffrant du VIH afin de dépister tôt les éventuels facteurs de risques.

## Conflits des intérêts

Les auteurs ne déclarent aucun conflit d’intérêts.
